# Imaging spectrum in rhino-orbito-cerebral mucormycosis: a cross-sectional study

**DOI:** 10.1097/MS9.0000000000001169

**Published:** 2023-08-10

**Authors:** Sharma Paudel, Pradeep R. Regmi, Prakash Kayastha, Shailendra Katuwal, Prasoon Ghimire, Suraj Shrestha, Urmila Gurung

**Affiliations:** a Department of Radiology; b Department of Otorhinolaryngology, Tribhuvan University Teaching Hospital; c Maharajgunj Medical Campus, Institute of Medicine, Kathmandu; d Department of Radiology, Dhaulagiri Hospital, Baglung, Nepal

**Keywords:** COVID-19, magnetic resonance imaging, rhino-orbital-cerebral mucormycosis

## Abstract

**Background:**

Rhino-orbital-cerebral mucormycosis (ROCM) is a life-threatening condition caused by a saprophytic fungus that predominately affecting immune-compromised patients. Early diagnosis of ROCM is of utmost importance to start the treatment as early as possible to prevent early and horrible complications of the disease.

**Materials and methods:**

This retrospective study evaluated the imaging findings of 21 patients with biopsy and KOH mount-based evidence of invasive ROCM. The imaging was obtained from a Siemens Magnetom Amira 1.5T system with a strength of 1.5T or more. The spectrum of findings was evaluated for the sites of involvement, signal intensity, contrast characteristics, necrotic component as well as orbital, infratemporal, and intracranial extensions, especially cavernous sinuses, Meckel’s cave, and the brain parenchyma.

**Results:**

The mean age of the patients was 55.8±10.9 years and included 71% male. All the patients were positive for COVID-19 and the majority were diabetic. MRI showed predominant involvement of the maxillary sinus (17, 81%) and the ethmoidal sinus (15, 71.4%). The orbital extension was present in 18 cases (86%). T1-weighted imaging showed iso to low signal intensity in involved sinuses in the majority of the patients (9, 42.9%). Heterogeneously high signal intensity was observed in T2-weighted and short tau inversion recovery images in all the patients. Heterogenous contrast enhancement was present in 20 (95.2%) patients.

**Conclusion:**

The imaging spectrum of ROCM is variable. Multiplanar MRI with postcontrast images is a very useful complementary tool to the clinical evaluation to assess the extent of disease and its complications, which has a high mortality. Clinicians and radiologists should be aware of the imaging spectrums of ROCM.

## Introduction

Highlights

- Rhino-orbital-cerebral mucormycosis is a life-threatening condition affecting immune-compromised patients.
- Ethmoidal and maxillary sinuses are commonly affected sinuses.
- T1-weighted iso to low signal intensity, heterogenous high signal intensity in T2-weighted and short tau inversion recovery images with heterogenous contrast enhancement is the most common MRI finding in rhino-orbital-cerebral mucormycosis.

Rhino-orbital-cerebral mucormycosis (ROCM) is a life-threatening condition caused by a saprophytic fungus that predominately affecting immune-compromised patients including diabetics, post-COVID status, and those on steroids. Mucor relates to the mucoracele family, which includes fungi like *Rhizopus* and *Absidia* ^1^. Mucors are distinct from aspergillosis as they are nonseptated and broad with right-angle branching^2,3^. Headache, fever, nasal obstruction, and orbital and facial pain are often the initial presentation of mucormycosis. It shows rapid progression with involvement of the cranial nerve and CNS involvement^4^. When brain invasion has occurred, mortality is greater than 80%. Because of its lethal nature, it must be recognized early and treated aggressively^5^. Thus, timely diagnosis of this condition is necessary, which can greatly alter the course of the disease. The purpose of this study was to describe the imaging findings of histologically diagnosed mucormycosis patients that will be useful in predicting the diagnosis of this dreadful infection. In addition, the outcome of these patients after surgical treatment was also reviewed.

## Materials and methods

### Study design

This is a descriptive, retrospective hospital-based study conducted at a tertiary care center, and a major referral hospital in Nepal. This study was conducted in a line with strengthening the reporting of cohort, cross-sectional, and case–control studies in surgery (STROCCS) criteria 2021^6^.

### Study population

**Figure 1:**
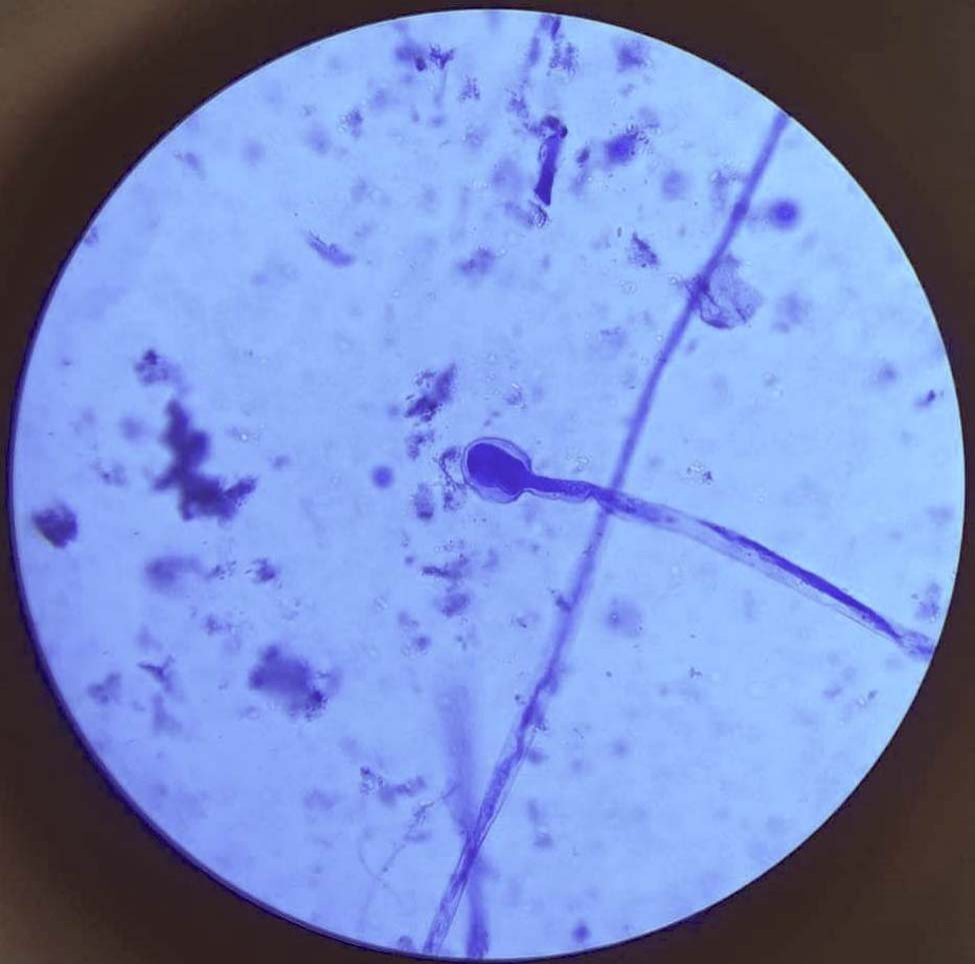
Broad, hyaline aseptate hypha on KOH mount.

We retrospectively evaluated the imaging findings of 21 patients with biopsy and KOH mount-based evidence of invasive ROCM with relevant clinical data. A KOH mount was prepared by adding a few drops of 10% KOH solution to the clinical specimen, mixed and incubated on the glass slide for 15 min, and examined under a microscope to identify the characteristic fungal elements such as nonseptate hyphae with right-angle branching (Fig. 1). Patients who underwent MRI with a Siemens Magnetom Amira 1.5T system with a magnetic strength 1.5T or more were included in the study. Participants with inadequate data of interest were excluded.

### Sample size

A total of 21 patients with evidence of invasive ROCM were included in the study. All eligible data of 1 year period (January 2021–January 2022) were considered for inclusion in the study.

### Data collection

The Axial, coronal, and sagittal images of T1-weighted (TR/TE/523/15ms), T2-weighted images (TR/TE/5430/109), T2 fat suppressed short tau inversion recovery images as well as T1-weighted images after intravenous injection of the nonionic macrocyclic agent, Gadobutrol (0.1 mmol/kg) were evaluated. Images were evaluated for the sites of involvement, signal intensity, contrast characteristics, necrotic component as well as orbital, infratemporal, and intracranial extensions, especially cavernous sinuses, Meckel’s cave, and brain parenchyma. The severity of ROCM was assessed using an MRI-based ROCM scoring system and divided into mild, moderate, and severe^7^. Clinical information as well as the outcome of the disease were obtained from the medical history in all cases. All the findings were recorded in a predesigned proforma and analyzed.

### Ethical approval

The ethical approval was obtained from the Institutional Review Committee of the hospital with an ethical approval number of 312 (6-11)E 2078/079 prior to the commencement of the study.

## Results

A total of 21 individuals were evaluated for this study with age ranging from 35 to 74 years. The mean age of the study population was 55.8±10.9 years. Among the study population, 71% (*n*=15) were male and 29% (*n*=6) were female. All the patients in the study tested positive for COVID-19 pneumonia by RT-PCR test. Among various comorbidities, diabetes mellitus was the commonest. Eighteen of twenty-one patients were diabetics, and only two of them had HbA1C level more than 6.5%. In addition, acute kidney injury with chronic kidney disease was present in three patients while chronic liver disease and hypertension were present in each of the study populations, respectively. A total of 19 patients (70%) received steroids as part of their treatment for COVID-19 pneumonia.

Among 21 patients who had undergone the KOH mount test, 19 of the patients were positive for fungal hyphae. Twenty out of twenty-one patients were positive for mucormycosis in the histological examination, including two of the patients who showed a negative fungal profile in the KOH mount test. One patient who was negative in histopathological findings for mucormycosis; however, showed broad and aseptate hyphae with single branching in the KOH mount test.

**Figure 2:**
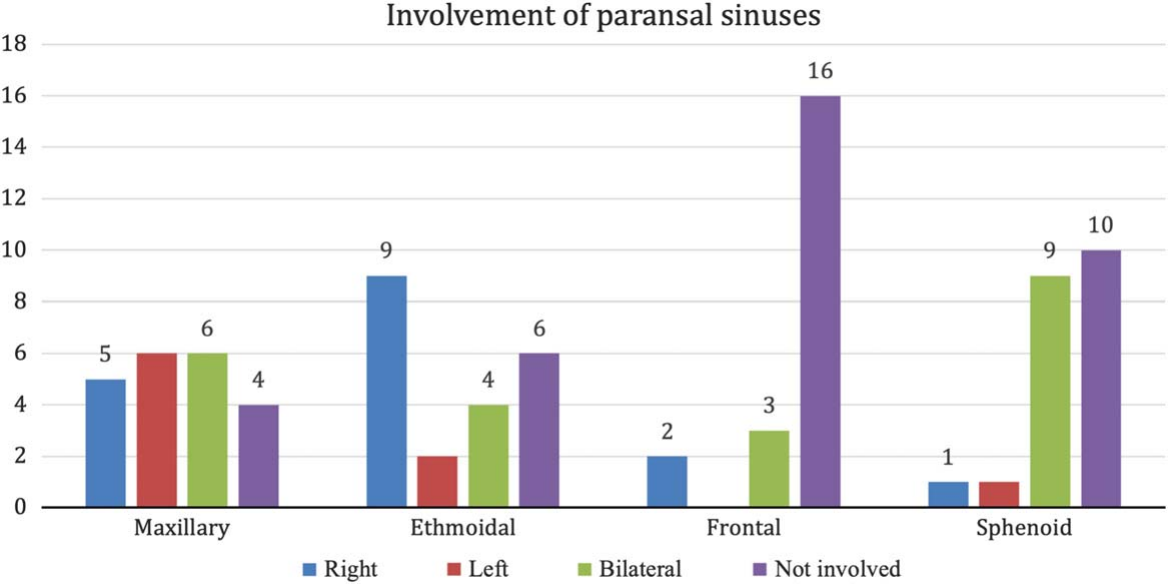
Bar graph showing various paranasal sinuses and their sides involvement.

Regarding the sinus involvement by mucormycosis, in our study, the involvement of the maxillary sinus was seen in 17 (81%) of the patients. Fifteen patients (71.4%) of them showed involvement of the ethmoidal sinus. Involvement of the frontal and sphenoid sinuses was seen in 5 (23.8%) and 11 (52.4%) patients, respectively. Among 21 patients, only 4 had isolated maxillary and 1 had isolated ethmoid sinus involvement. Most of the patients had involvement of multiple sinuses (Table 1). Eight patients in our study showed involvement of more than two paranasal sinuses (PNS) (Fig. 2).

**Table 1:** Paranasal sinus involvement in each individual.

Sinuses involved	Frequency (*n*=21)
Isolated maxillary	4
Isolated ethmoid	1
Maxillary +ethmoid	4
Ethmoid + sphenoid	2
Maxillary + sphenoid	2
Pansinusitis	8

MRI images with contrast were obtained in all the study populations. We observed T1-weighted iso to low signal intensity in involved sinuses in most of the patients, 9 (42.9%) out of 21 patients. In addition, low, iso, and iso to high signal intensity were observed in 6 (28.6%), 4 (19.0%), and 2 (9.5%) patients, respectively. However, heterogeneously high signal intensity was observed in T2-weighted and short tau inversion recovery images in all the patients. In terms of contrast enhancement, heterogenous enhancement was present in 20 (95.2%) of the patients, and only 1 (4.8%) had homogenous enhancement (Table 2).

**Table 2:** MR signal intensity, contrast enhancement, and bony sinus wall involvement.

MR characteristics	Frequency (*n=*21)	Percentage (%)
T1 W signal intensity		
Iso to low	9	42.9
Iso to high	2	9.5
Low	6	28.6
Iso	4	19.0
T2 W signal intensity		
Heterogeneously high	21	100.0
STIR signal intensity		
Heterogeneously high	21	100.0
Contrast enhancement		
Heterogenous	20	95.2
Homogenous	1	4.8
Necrotic area		
Present	13	61.9
Not present	8	38.1
Bony sinus wall erosion		
Present	12	57.1
Not present	9	42.9

STIR, short tau inversion recovery.

**Figure 3:**
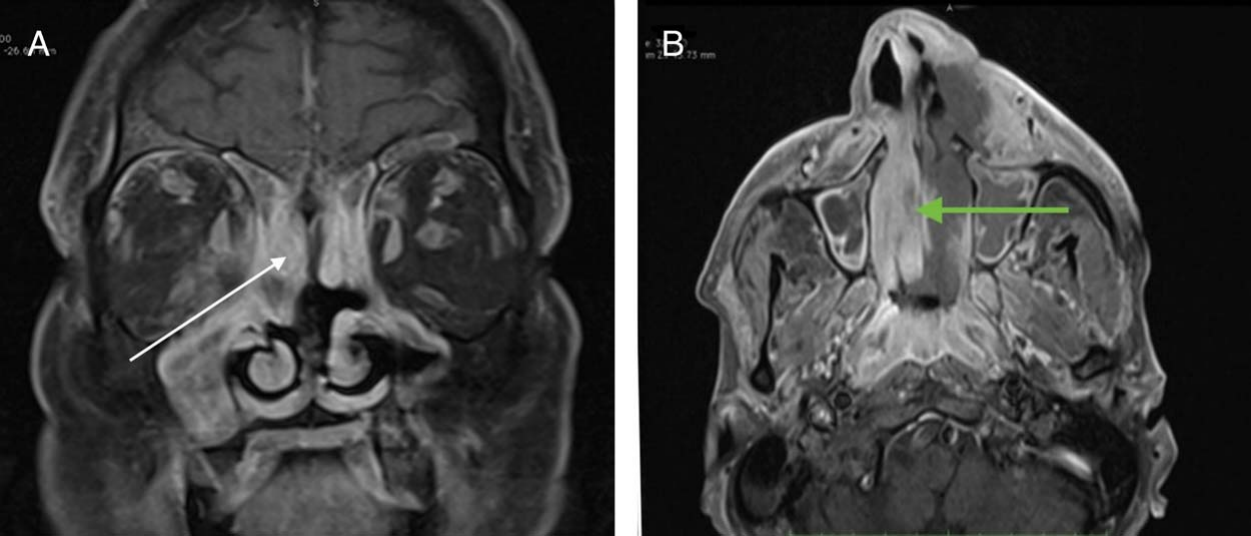
(A) Heterogeneously enhancing thickening in bilateral maxillary, and ethmoidal sinus (white arrows). (B)Heterogeneously enhancing soft tissue thickening with swelling in left premaxillary subcutaneous plane. Nonenhancing necrotic component (green arrow) seen in left middle turbinate, nasal septum, dorsum and left lateral surface of nose and left premaxillary subcutaneous plane.

**Figure 4:**
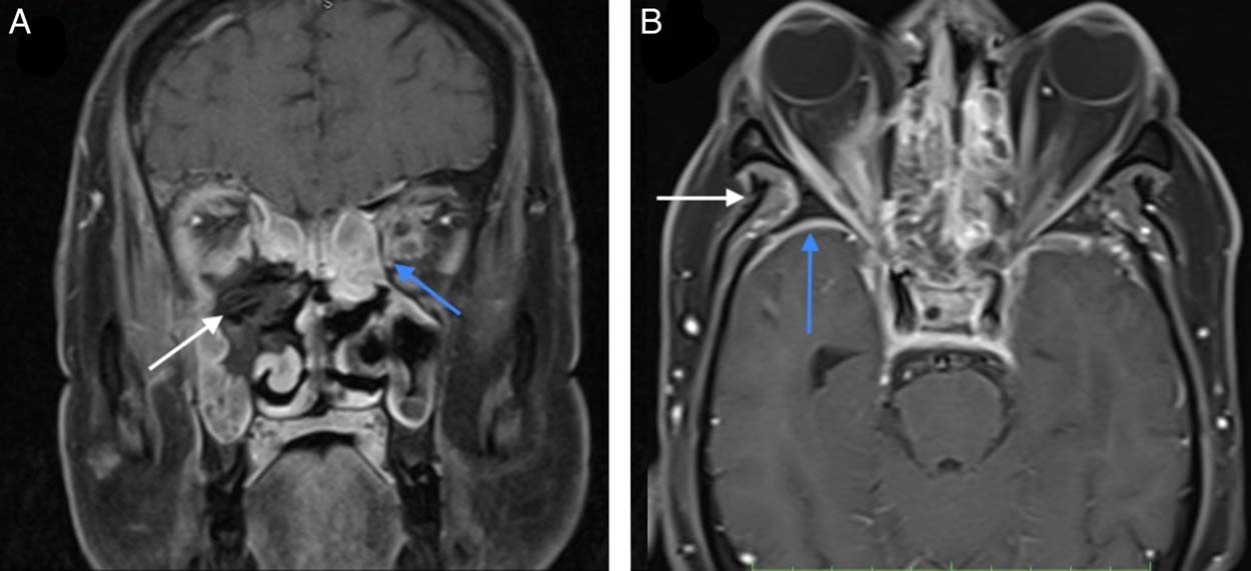
65-year-old male with post-COVID status and blackish nasal discharge. (A) Necrotic component (white arrow) involving right ethmoidal sinus, right middle turbinate, medial and inferior wall of right orbit with erosion of wall. Perioptic strandings (blue arrow) is seen in left orbit. (B) Thickened enhancing dura (blue arrow) in right temporal lobe region with enhancing inflammatory changes (white arrow) in right infratemporal fossa.

There was the presence of necrotic areas in 13 (61.9%) of the cases (Fig. 3). Necrotic areas were predominantly observed within the PNS; however, it was seen extending to the premaxillary region and orbits in two individuals. Erosion of bony sinus walls was seen in 12 (57.1%) cases with half of them showing erosion of the wall of the maxillary sinus (Table 2). Among 21 individuals, orbital involvement was seen in 18 cases (86%). Of them, seven (38.9%) showed erosion of the orbital wall. Extraconal involvement was observed in 15 (83.3%) patients in whom the predominant finding was enhancement and swelling of the extra-ocular muscles with surrounding inflammatory changes. Intraconal involvement was seen in seven (38.9%) patients and optic nerve involvement were seen in one patient (Fig. 4). Seven individuals (38.9%) also showed orbital apex involvement.

**Figure 5:**
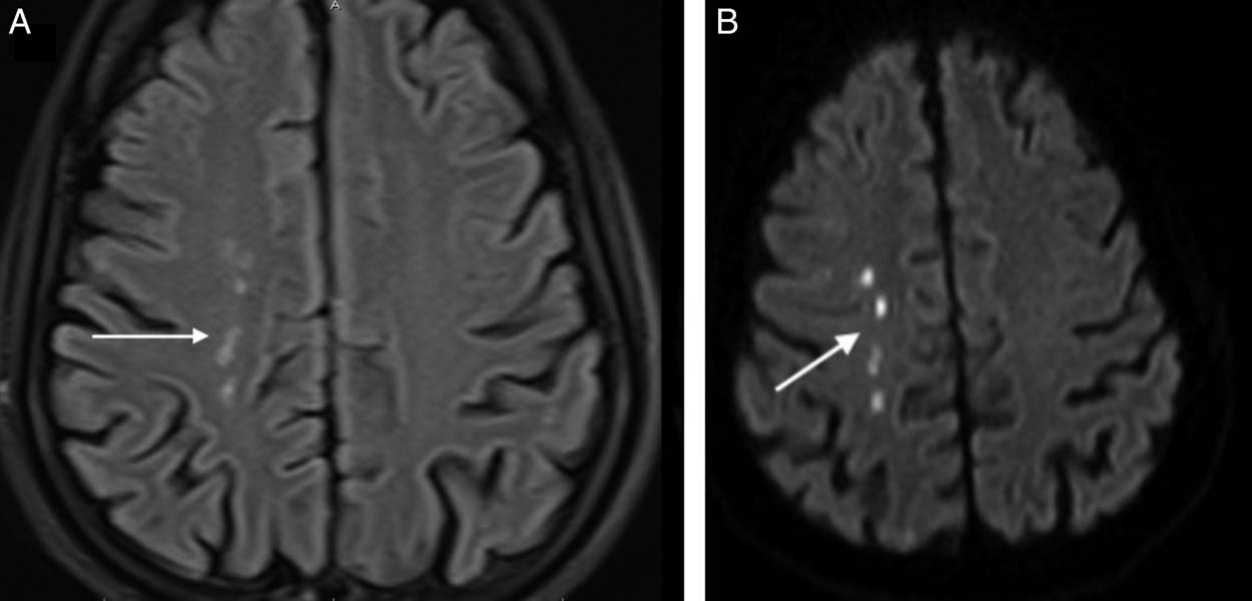
A 55-year-old COVID-19 positive male presenting with ptosis. (A) FLAIR axial image showing multiple flair high signal intensity (white arrow) in watershed region of right anterior cerebral and middle cerebral artery. (B) DWI image of the same patient shows restriction of these lesions (white arrow) suggestive of multifocal infarct.

**Figure 6:**
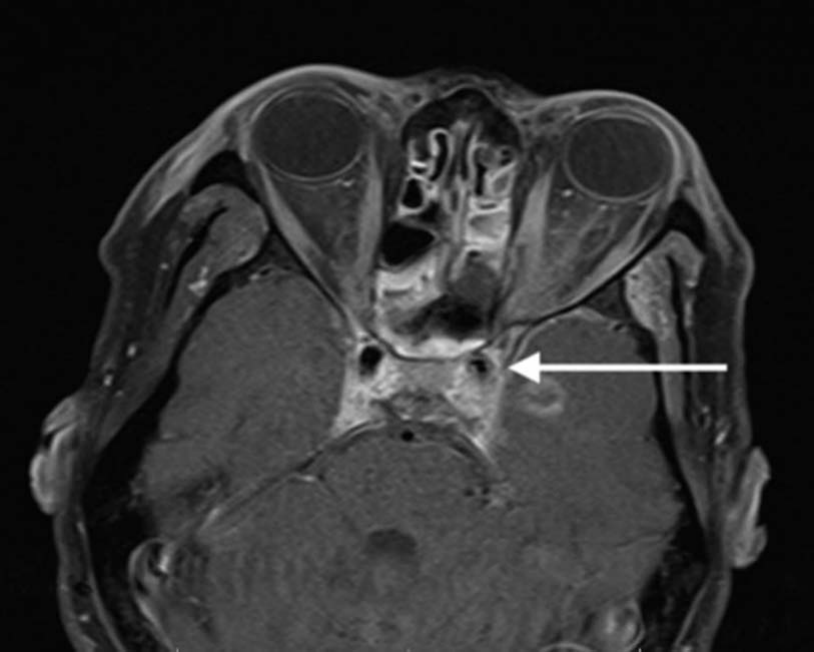
A 48-year-old male presenting with the mild left sided facial numbness and sinusitis. Axial postcontrast T1-weighted image shows heterogeneously enhancing increased signal intensity in the left intraconal space with extension to left cavernous sinus causing luminal narrowing of cavernous segment of left internal carotid artery (white arrow). Bilateral dural enhancement of cavernous sinus and enhancing parenchymal lesion in left temporal lobe is seen.

In cases with intracranial involvement, the most commonly observed finding was meningeal/dural enhancement, which was seen in eight cases (38.1%). In these cases, the predominant finding was dural thickening and enhancement along the anteromedial and inferomedial convexity of the temporal lobes. Cavernous sinus and brain parenchymal involvement along with hematoma and vascular involvement were also observed (Figs 5 and 6). Extension to Meckel’s cave was seen in two cases (9.5%) (Table 3).

**Table 3:** Frequency distribution of involvement of extrasinus and extraorbital structures.

Extrasinus and extraorbital involvement	Frequency (*n*=21)	Percentage (%)
Intracranial involvement		
Cavernous sinus	7	33.3
Meckel’s cave	2	9.5
Meningeal/Dural enhancement	8	38.1
Brain parenchyma	3	14.3
Vascular	3	14.3
Infratemporal Fossa	17	81.0
Premaxillary/Periorbital soft tissue	12	57.1
Palate	2	9.5
Others	6	28.6

**Figure 7:**
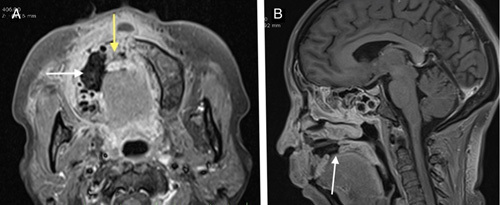
Young diabetic COVID-19 positive male with right nasolabial swelling. (A) Defect in the alveolar process of right maxillae with nonenhancing component (white arrow). Enhancing marrow in incisor part of maxilla (yellow arrow) with premaxillary soft tissue swelling. (B) Defect in the hard palate (white arrow).

The infratemporal fossa was the most common surrounding structure involved by mucormycosis. as observed in 17 patients (81%). Among these patients, there was also an extension to masticator space in seven cases. Premaxillary/periorbital swelling was seen in 12 cases (57.1%). Involvement of the palate, alveolar process, clivus, nasopharynx, and dorsum of the nose were other affected structures (Fig. 7). Extra-axial nonenhancing collection in the right anterior temporal lobe convexity was observed in one of the patients as well. Staging was done based on ROCM scoring for MRI imaging findings of rhino-orbito-cerebral mucormycosis, which showed six (28.5%) of patient in the mild stage, seven (33.4%) in moderate stage, and the remaining eight (38.1%) in the severe stage of the disease (Table 3).

## Discussion

Paulltauf was the first to describe mucormycosis in 1885, also known as phycomycetes^8^. The fungal spores of phycomycetes are found freely in the soil, skin, dust, and spoiled food. These spores are inhaled which become pathogenic in immune-compromised patients including poorly controlled diabetic and diabetic ketoacidosis, hematological malignancy, and chronic kidney disease. The disease initially involves the nasal cavity and paranasal sinus, which later extend to extrasinus sites including the orbit and brain. Involvement of orbit is via the nasolacrimal duct and medial orbital wall. It also invades the vessels causing thrombosis with infarction and dissemination to brain parenchyma. Based on the anatomical site of involvement mucormycosis is classified into six different types: (1) rhino cerebral, (2) pulmonary, (3) cutaneous, (4) gastrointestinal, (5) disseminated, and (6) uncommon presentations^9^.

Immunosuppressed status, predominately diabetes was found in 87.5% of the patients with COVID-19-associated mucormycosis in a study conducted by Yogendra *et al*.^10^. In our study conducted on 21 patients with COVID-19 and mucormycosis, 85.7% were found to be diabetic. Although there is existing evidence linking COVID-19 to mucormycosis, pinpointing the actual cause and effect is challenging due to the existence of two significant confounders, diabetes, and indiscriminate steroid usage. The SARS-CoV-2 virus has been linked to the disruption of the cell-mediated immune response that results in mucormycosis invasion, tissue necrosis, and thrombosis^11^.

Bhansali *et al*., Ferry *et al*., and Yohai *et al*. reported 100, 69, and 79% of the PNS involvement in ROCM, respectively^12–14^. In a study conducted by Sharma *et al*.^15^ ethmoidal sinus was most commonly involved (100%). In our study, most of the patients showed the involvement of multiple sinuses. However, maxillary and ethmoidal sinuses were the most common. Maxillary sinus was involved in 81% of the patients.

Aggressive involvement of the sinonasal and orbital cavities by mucormycosis was shown by Herrera *et al*.^5^. Orbital involvement is mostly by retrograde spread along the nasolacrimal duct and through the lamina papyraceae^12^. In our study, 86% of the cases showed orbital involvement with wall erosion in 38.9% of the cases. Mucormycosis is the most important fungal infection for orbital apex syndrome which occurs through the sphenoid sinus and is an important conduit for intracranial spread^16^. In our study, out of seven patients with orbital apex involvement, concomitant bilateral sphenoid sinus involvement was seen in five (71.42%) patients.

In MRI, the largest number of patients showed iso to low signal intensity with the less common signal being low and high signal intensity. All the patients showed T2 heterogenous high signal intensity with areas of low signal intensity. The presence of manganese and iron in hyphae is the reason for the low signal changes^3^. Postcontrast study with Gadolinium showed mostly heterogeneous enhancement. Only one of the patients showed a homogenous enhancement. In a study done by Upender *et al*.^17^, 50% of the patients showed the presence of nonenhancing necrotic tissue. Our study showed about 62% of necrotic/nonenhancing tissue predominately in the sinuses, premaxillary region, and orbit.

Vascular involvement in invasive mucormycosis is a direful complication with poor prognosis. Mazzai *et al*.^18^ in their meta-analysis of 42 cases with vascular complications in rhino cerebral mucormycosis studied between 1975 and 2019, arterial and/or cavernous sinus thrombosis was seen in 35 cases with cerebral ischemia, subarachnoid hemorrhage in 6 cases, 5 cases of an arterial aneurysm, and septic emboli in 1 case.

The spread of mucormycosis from the orbit and orbital apex involves the cavernous sinus, which leads to thrombosis with loss of flow void and convex margin of the sinus^12^. Mucormycosis has a high propensity for arterial invasion as it reproduces in the internal elastic layer of vessels causing separation of the elastic and middle layers with intimal damage and thrombosis^19^. Cerebral infarction due to purulent arteritis and inflammatory emboli was reported by Ma *et al*.^20^. Eucker *et al*.^21^ reported mycotic cerebral vasculitis and vascular occlusion as a leading cause of cerebral infarction. Brain parenchymal involvement in the form of multifocal infarct and hematoma was seen in 14.3% of patients, which is similar to the study performed by Jacob *et al*.^3^. Involvement of cavernous segment of the internal carotid artery with attenuated caliber was seen in 14.3% of patients which is one of the leading causes of cerebral infarction. Our study showed the involvement of dura/meninges in 38.1% as a thickening and enhancement predominately involving the anteromedial and inferomedial convexity of temporal lobes. Similar results were obtained by Desai *et al*.^22^ (42%) in a study conducted in 50 patients with mucormycosis. In another study conducted in five patients, 60% of dural enhancement and thickening was noted which is higher than our study^5^. It may be due to the small size of the sample.

Mucormycosis can show the retrograde perineural spread along the trigeminal nerve involving the cavernous sinus and the Meckel cave causing its widening^23^. In our study, two patients had Meckel’s cave involvement where a cavernous sinus of the same side was involved. Only one patient was associated with hemifacial numbness on the same side of Meckel’s cave involvement. The extracranial, extraorbital, and extrasinus involvement were most seen in the infratemporal fossa (81%) followed by premaxillary soft tissue in the form of soft tissue signal intensity.

Early diagnosis of ROCM is of utmost importance to start the treatment as early as possible to prevent early and horrible complications of the disease. The various methods used for the diagnosis of mucormycosis are KOH preparation, histopathological, and culture studies^24^. KOH mount preparation is highly sensitive (64%) with a high negative predictive value of 94.23% while histopathology is highly specific (96.3%)^25^. In our study, 90.5% of patients show positive for the fungal hyphae in KOH mount, and 95.2% positive for mucormycosis in histopathology. Two patients who were negative for mucormycosis in KOH mount were positive in histopathology and one patient negative in histopathology was positive in KOH mount. These differences observed between the examination findings could be because the samples sent to the microbiology department for KOH and histopathology may not be from the same site and hence may not be representative. Additionally, the tissue section may be scanty or taken from a deep section or very superficial section^26,27^. In one of the patients, Aspergillus was identified in KOH and histopathology in addition to the mucormycosis.

Various factors like anatomical involvement (nasal, orbital, and nervous system), presence of comorbidities, and presence of effective treatment affect the prognosis of ROCM^17^. ROCM has a high mortality rate of ~45% in patients with diabetes mellitus and 35% in patients without any underlying medical conditions^28^. A multidisciplinary approach with early appropriate surgical and systemic antifungal therapy reduced the mortality from 88% in 1961 to the current 15–35%^29^. According to Ericsson *et al*.^30^, 80% of patients with ROCM receiving timely medical and surgical care survived. The mortality rate is still high in patients with CNS involvement in initial examination^31^.

In our study, one patient died due to ROCM, who had the imaging finding of involvement of the internal carotid artery, brain parenchyma, cavernous sinus, Meckel’s cave, and orbital apex. The mortality data could be higher in our study; however, long-term follow-up could not be done.

There are a few limitations to this study. A relatively small number of cases were included in the study. A study comprising a larger number of patients would further substantiate the results of the study. Since this is a retrospective study, there is a possibility of incomplete evaluation of patients leading to the underestimation or overestimation of the severity distribution across different stages and lack of other necessary information. In addition, a long-term follow-up of the patients could not be performed.

## Conclusion

The imaging spectrum of ROCM is variable. Particularly common in immunosuppressed and diabetic patients with positive COVID-19 status, heterogeneous signal intensity showing heterogenous enhancement and nonenhancing necrotic content in MRI are seen in different sinuses. Patients in the advanced stage show an aggressive extension to the orbital, intracranial as well as other extrasinus regions. Multiplanar MRI with postcontrast images is a very useful complementary tool to clinical evaluation to assess the extent of disease and its complications. Despite aggressive surgical and medical management, ROCM carries the worse prognosis with a higher mortality rate. Therefore, clinicians and radiologists should be aware of the imaging spectrums of the disease.

## Ethical approval

Ethical approval was received from the Institutional Review Committee of Institute of Medicine, Maharajgunj, Kathmandu, Nepal on 3 February 2022 prior to the start of this research. The reference number of the approval is 312 (6-11)E 2078/079.

## Consent

Since this was a retrospective study, the requirement for informed consent from the study participants was waived.

## Sources of funding

The study has no financial assistance of any kind.

## Author contribution

S.P.: conceptualization, data curation, methodology, supervision, validation, writing – review and editing; P.R.R.: conceptualization, data curation, methodology, writing – original draft, writing – review and editing; P.K.: writing – review and editing; S.K.: conceptualization, data curation, methodology, writing – original draft, writing – review and editing; P.G.: data curation, writing – original draft, writing – review and editing; S.S.: writing – original draft, writing – review and editing; U.G.: supervision, validation, writing – review and editing.

## Conflicts of interest disclosure

The authors declare that they have no conflicts of interest.

## Research registration unique identifying number (UIN)

1. Name of the registry: not applicable.
2. Unique identifying number or registration ID: not applicable.
3. Hyperlink to your specific registration (must be publicly accessible and will be checked): not applicable.

## Guarantor

Dr Pradeep Raj Regmi. E-mails: pradeepregmi@iom.edu.np, pradeep.iom@gmail.com.

## Data availability statement

All the necessary information is provided within the manuscript. Additional tables, figures, and STROCCS checklists have been supplied as supplementary files.

## Provenance and peer review

Not commissioned, externally peer-reviewed.
